# Thirty Years Later: Pregnancies in Females Perinatally Infected with Human Immunodeficiency Virus-1

**DOI:** 10.1155/2012/418630

**Published:** 2012-08-28

**Authors:** Martina L. Badell, Michael Lindsay

**Affiliations:** Department of Gynecology and Obstetrics, Emory University, 69 Jesse Hill Jr. Dr. S.E., Atlanta, GA 30303, USA

## Abstract

The first cases of mother to child transmission of human immunodeficiency virus (HIV) were described more than two decades ago and since then several thousands more have been reported in western countries. In the early 1980s the majority of perinatally acquired HIV children did not survive beyond childhood. However combined antiretroviral therapy (ART) for perinatally HIV-acquired children has prolonged their survival and in the past 2 decades, many have reached adulthood. As the perinatally HIV-infected females become sexually active, they are in turn at risk for pregnancy and of transmitting HIV infection to their children. A considerable proportion of this population appears to engage in unprotected sexual intercourse leading to teenage pregnancies, STDs, and abnormal cervical cytology despite frequent contact with HIV health care providers and clinics. Currently there is a paucity of data regarding pregnancy and neonatal outcomes in HIV perinatally infected women. As increasing number of pregnancies will occur among this population we must continue to monitor and focus on their reproductive health issues to improve perinatal and long-term maternal outcomes. This paper will summarize our current knowledge about reproductive health issues and identify areas for future inquiry.

## 1. Introduction

More wide scale HIV testing and counseling along with progress in HIV treatment has led to major clinical advances in HIV care and has transformed HIV/AIDS from a uniformly fatal disease to a chronic disease. According to UNAIDS program, 33 million people worldwide were estimated to be living with HIV or AIDS at the end of 2009 [1]. The majority of infected individuals are adults and reside in the developing world.

During the early 1980s when the first perinatally acquired AIDS cases were documented, infection usually progressed rapidly to death. In the United States and other developed nations through public health strategies that emphasized prenatal HIV screening and use of ART the number of perinatal HIV cases have decreased dramatically from 1,650 in 1991 to fewer than 200 in 2004 which represents an overall 92% decline [2–5]. In 2005, the estimated number of perinatally infected persons living with HIV was 6,051 for the 33 jurisdictions with HIV reporting in the United States [6]. As the perinatally infected cohort have benefited from antiretroviral therapy, there has been a significant decrease in pediatric AIDS deaths. Thus, perinatally infected children are living longer and the first wave is now approaching adolescence and young adulthood. A female is considered to have perinatally acquired HIV infection if her mother was HIV infected during pregnancy, labor or delivery according to clinical records or if she is found to be positive during infancy or early childhood without another explanation for exposure [7].

Health care providers of perinatally infected young women of reproductive age are now encountering reproductive health issues in this population with little or no evidence to guide them. Adolescents perinatally infected with HIV are often cared for in pediatric infectious disease clinics where reproductive health issues may not be routinely addressed. Several studies have examined reproductive health issues in this population [8–10]. One study found that 70% expressed intent to have children [9] and many demonstrated limited knowledge of safe sex practices [10]. In an editorial response to the first report describing pregnancy in perinatally HIV-infected adolescents and young adults, the Centers for Disease Control recommended enhanced efforts to identify pregnancies among this population and more in-depth investigation of such pregnancies to better characterize the factors associated with these pregnancies and their outcomes [11]. Since 1998, 13 reports of pregnancies among perinatally infected adolescents have been described [7, 9, 11–21]. This paper will summarize our current knowledge about pregnancies in this special population and identify areas for future inquiry. 

## 2. Pregnancy Outcomes

### 2.1. Preterm Birth

The first case report of pregnancy in a perinatally HIV infected female was described in 1998. The patient was a 14-year old who delivered an HIV negative infant at term [12]. However, perinatally HIV infected females appear to be at increased risk of preterm birth. In 2009, Williams et al. published a retrospective review of maternal and neonatal outcomes of 10 perinatally HIV-infected females [19]. They found the median age of first pregnancy was 18.5 years and the mean gestational age at the time of delivery was 38 weeks. However, premature rupture of membranes with preterm delivery occurred in 31% of the patients. Teenage pregnancy is a known risk factor for preterm birth with rates reported among adolescents between 13–18% [22, 23]. The rate in this perinatally HIV-infected cohort is significantly higher than the expected risk from adolescence alone. In addition, Thorne et al. evaluated nine viable pregnancies in perinatally HIV-infected females from Europe and found preterm delivery occurred in 44% [17]. More recently Beckerman et al. found perinatally infected females (*n* = 34) compared to sexually infected women *n* = 54 were significantly more likely to delivery prematurely with a mean gestational age of 33.7 versus 38.8 weeks *P* = .03 [21]. The etiology of the increased risk for preterm delivery in this population is unclear. Early data is conflicting as to whether receipt of combination ART during pregnancy is associated with preterm delivery [24]. A recent pooled analysis of three large studies found heterogeneity in the association between combination ART and preterm birth. However, increased rates of preterm birth (adjusted OR 1.5) were found in all three cohorts when combination ART regimens were compared with dual regimens. Additional factors found to be associated with preterm birth in all three cohorts included injection drug use and more advanced HIV disease [25]. Clinicians should be aware of a possible small increased risk of preterm birth in pregnant women receiving protease inhibitor (PI)-based combination ART; however, given the clear benefits of such regimens for both the women's health and the prevention of mother-to-child transmission (MTCT), providers should not withhold protease inhibitors for fear of altering pregnancy outcome [24]. 

Premature delivery in perinatally HIV infected females has an impact on the selection of ART for the newborn. Prematurity is associated with increased mortality and a number of neonatal morbidities including respiratory distress syndrome, intraventricular hemorrhage, and necrotizing enterocolitis. In premature infants medication dosing is only available for zidovudine therefore making the use of other ART more problematic. Preterm infants' immature renal and hepatic metabolism increases the risk of ART overdosing and toxicity. Postmarketing surveillance identified 10 neonates, 9 of whom were born prematurely, who received lopinavir/ritonavir and experienced life-threatening events [26]. Due to these increased adverse effects predominantly seen in preterm neonates, the Food and Drug Administration now recommends that lopinavir/ritonavir not be administered to neonates before a postmenstrual age of 42 weeks and a postnatal age of at least 14 days [24]. Finally, premature infants may be at increased risk for viral resistance secondary to a short duration and amount of ART exposure in the mother. Thus, premature delivery in this population has both immediate and long term adverse consequences and may compromise the clinician's ability to provide state of the art ART to the infant to prevent MTCT.

### 2.2. Cesarean Delivery

Perinatally infected females appear to be at increased risk of delivering by cesarean section. In the United States, a scheduled cesarean delivery (CD) at 38 weeks of gestation to prevent perinatal transmission of HIV is recommended for all HIV-infected women if their plasma HIV RNA remains >1,000 copies/mL near the time of delivery [24]. The rate of CD in the United States in 2007 for women under 20 was 23% [27]. In the perinatally infected cohort described by Williams et al., the rate of CD was 62% with 75% of the cases performed for HIV infection [19]. Inadequate viral suppression in that cohort reinforces the need for close prenatal followup and explicit counseling on the importance of medication adherence. In a European study of 9 viable pregnancies in perinatally HIV-infected females, 8 of the 9 (89%) were delivered by CD [17]. Six of these were described as elective and the other 2 were emergency CD. Details surrounding the decision for mode of delivery was not described, however, 2 of the 8 patients who underwent CD had a viral load >1000 copies/mL at the time of birth. Compared to vaginal birth, CD are associated with increased morbidity including excessive blood loss, infection, thromboembolism, and postoperative pain. In addition, repeat CDs have additional risks of operative injuries and of abnormal placentation including placenta previa and placenta accreta. Given the young age of these patients and potential likelihood of future pregnancies the optimal delivery would include viral suppression from adequate ART leading to opportunities for vaginal birth. 

In contrast to the retrospective data from the United States, a prospective study of 30 asymptomatic perinatally HIV-infected adolescents and young adult females in India found no risk of adverse maternal or fetal outcome. The study revealed a low rate of preterm birth (3%) and cesarean delivery (3%) [13]. However the results of this study may in part be explained by selection bias as this study population included perinatally HIV-infected females who had an absence of STDs, received regular medical care, ART, had good nutrition, and were mostly married with a strong desire for pregnancy. In addition, any clinical manifestation of HIV disease was an exclusion criterion for this study. Therefore, although these results are somewhat reassuring, they may not be generalizable to other perinatally HIV-infected female populations.

## 3. Perinatal HIV Transmission

Before the introduction of ART and obstetrical interventions to reduce MTCT about 1 in 4 infants born to a woman infected with HIV became infected. Among infected infants approximately 50% of transmission occurs around the time of labor or delivery, 20–25% occurs in-utero, and 25–35% occurs postnatally secondary to breastfeeding [28]. In developed countries today, MTCT rate are estimated at less than 2% with the use of ART during pregnancy and in labor, with cesarean deliveries for viral loads >1,000 (copies/mL), 6 weeks of neonatal ART prophylaxis and avoidance of breastfeeding [4, 29]. Although ART has markedly decreased the risk of MTCT in the United States among adult females infected with HIV, little is known about their effect among pregnant perinatallyinfected females.

The risk of MTCT among perinatally HIV infected females appears to be comparable to the MTCT among nonperinatally HIV infected parturients. The Pediatric AIDS Clinical Trials (PACTG) protocol 219 has enrolled and followed HIV-infected and non-HIV-infected children at clinical centers across the United States since September 2000 to study the complications of pediatric HIV infection. A subanalysis including only perinatally infected adolescent girls aged 13 or older identified a cohort of 638 adolescent girls in which there were 32 pregnancies resulting in live births. One infant was HIV infected, 29 were uninfected, and 2 had unknown infection status, for a rate of MTCT of 3.3% (95% CI = 0.1, 18.6) [7]. All adolescent girls received ART during pregnancy with 26 receiving combination therapy with at least 3 drugs including a protease inhibitor (PI); the case of perinatal transmission occurred in 1 of the 2 girls receiving a PI and a nonnucleoside reverse transcriptase inhibitor (NNRTI). In the cohort described by Williams et al., there was 1 case (10%) of perinatal HIV transmission however this was attributed to patient noncompliance [19]. At the time of publication of the European study, one of the nine infants was confirmed uninfected, seven were presumed uninfected and the most recently born was still indeterminate [17]. Although this corresponds to a 0% MTCT the 95% confidence interval is wide because of the small sample size and the upper limit is 36.7%. An earlier study from Puerto Rico identified eight cases of pregnancy in perinatally HIV-infected females. These resulted in six viable infants with no MTCT at the time of publication [11]. Most recently Millery et al. reported on 19 live births in a cohort of perinatally infected women from New York City in which there were no cases of MTCT [20]. Young people may have problems adhering to ART and pregnancy could compound this [30, 31]. Therefore, young infected pregnant women need additional counseling about proper ART use and social support to achieve maximal viral suppression which is vitally important to preventing MTCT. 

## 4. Other Reproductive Health Concerns

### 4.1. Sexually Transmitted Infections

High risk sexual behavior is a concern in perinatally HIV-infected females. In the PACTG 219 cohort of perinatally infected adolescent girls, there was a six year cumulative incidence of Trichomoniasis of 6.8% (95% CI 2.4–11.5) and Chlamydia of 5.5% (95% CI 2.0–9.1) [6]. These rates are lower than (6–22%) documented in the REACH cohort a prospective cohort of 330 HIV-positive homeless and marginally housed persons recruited in San Francisco. However, that cohort included girls infected with HIV through sexual contact [32]. Also screening for genital infections was not performed routinely as part of the PACTG 219 protocol. The rate of sexually transmitted infections (80%) was significantly higher in the ten pregnant perinatally infected adolescents reviewed by Williams et al. [19]. These findings of a high STD rate among perinatally HIV-infected females underscore the importance of obtaining sexual histories, promoting safer sexual practices and screening for STDs in pregnancy in this population.

### 4.2. Cervical Cytological Abnormalities

According to the most recent guidelines cervical cancer screening should begin at age 21 years [33]. Women infected with HIV are at an increased risk of high-risk human papillomavirus (HPV) infection and cervical intraepithelial neoplasia (CIN) [34–36]. Although adolescents with HIV have a higher incidence of cervical dysplasia, the incidence of high-grade abnormalities (both high-grade squamous intraepithelial lesion and CIN 2 or CIN 3) appears to be low [37, 38]. Therefore, cytologic screening in this population is recommended twice in the first year after diagnosis and annually thereafter, with referral for colposcopy for any cytologic abnormality other than atypical squamous cells of undetermined significance (ASC-US) [39, 40]. The current guidelines do not specifically address when to begin screening in a patient with perinatally infected HIV. However, it would seem prudent to begin screening at the onset of sexual activity and certainly at the time of prenatal care in the absence of cytology screening in the previous 12 months.

In the PACTG 219 cohort, 48 of 101 girls (47.5%) with a Papanicolaou smear (Pap smear) had abnormal cervical cytology, including ASCUS (*n* = 18), low-grade squamous intraepithelial lesions (SIL) (*n* = 27), and high-grade SIL (*n* = 3) [7]. The mean age at first Pap smear was 16.7. Among the 21 adolescent girls who underwent intervention 10 (48%) persisted or progressed to more severe lesions. In the Williams et al. review of 10 pregnant patients with perinatally HIV infection 5 of 10 (50%) had abnormal cervical cytology [19]. The high proportion of abnormal cervical cytology in these adolescent girls is likely secondary to an increased susceptibility to and persistence of HPV and other genital infections as has been documented in nonperinatally HIV-infected females [41]. 

Finally, an equally important public health issue in perinatally HIV-infected young females is the use of the HPV vaccine. There currently are no guidelines which specifically address the use of HPV vaccine in this population. Although safety of the quadrivalent vaccine in HIV-infected children has been demonstrated, efficacy in women or girls with HIV has not yet been established [42]. HIV is not considered a contraindication to receiving the vaccine and at this time the CDC recommendations for HPV vaccination of children and adolescents should be followed similarly for both HIV-infected and non-HIV-infected populations [39, 43, 44].

## 5. Compliance with Treatment

Adherence to antiretroviral therapy is poorer during adolescence in HIV-infected individuals compared to younger children and adults [45]. Poor compliance with medications and care impacts significantly on virologic control. Adolescence is known to be a time of increased risk taking behaviors and the impact on adherence has been documented in other chronic health conditions such as cystic fibrosis [46]. Young people with HIV have the additional burden of stigma, secrecy, and the risk of transmitting HIV to partners and offspring. Perinatally HIV-infected females have multiple barriers to adherence including mental health/substance abuse, low expectancy for outcome of ART, and structural barriers in fitting medication into a complicated daily life [47]. In addition, many perinatally HIV-infected females have suffered bereavement losing their mothers which impacts their adult support network and possibility leaving them to care for other family members. As adolescences is a time often associated with poorer adherence to medications, adherence messages need to be repeated and particular attention should be given to the period during the transition from pediatric to adult services [48]. Counseling regarding family planning options and providing easy access to contraception is also of vital importance. The cohort of 15–25 year old perinatally infected patients in New York City identified 33 total pregnancies among 96 women of which fourteen (48%) were electively terminated [21]. In order to proactively reduce the risk of undesired pregnancy we must provide education and counseling on sexuality, reproductive health, and contraception. By focusing more efforts on this high-risk patient population at this important transition time in their lives we may achieve the goal of decreasing risk taking behaviors leading to unprotected sex, STDs, unintended pregnancies and noncompliance with ART and followup. 

## 6. Conclusions

As the perinatally HIV-infected female population ages increasing numbers of pregnancies can be anticipated and reproductive health issues affecting this population will need to be addressed. A considerable proportion of this population appear to engage in unprotected sexual intercourse leading to teenage pregnancies, STDs, and abnormal cervical cytology despite frequent contact with HIV clinics. Although there is a paucity of published data on this population, the findings to date highlight the importance of obtaining sexual histories, counseling to prevent unintended pregnancies, screening for STDs, and performing routine Pap smears. In addition, further studies are needed to accurately assess the maternal, obstetrical, and neonatal risks associated with pregnancy in perinatally HIV-infected females. MTCT rates are now less than 2% in the United States when pregnant women with HIV infection receive ART, undergo elective cesarean delivery, and refrain from breastfeeding. Based on the current limited evidence it appears that the MTCT rate is similar in perinatally HIV-infected females compared to nonperinatally HIV-infected parturients. The rate of preterm birth and cesarean deliveries appears to be higher in perinatally HIV-infected females however the overall small sample sizes currently precludes determining an accurate relative risk. If the current treatment advances in HIV infection continue we anticipate identification of additional pregnancies in perinatally HIV infected females. Future research should focus on identifying prospective cohorts and characterizing pregnancy outcome, rate of MTCT, determinants of contraceptive choices, and HIV disease progression. We must continue to monitor and focus on the reproductive health issues in this population to better understand and improve perinatal and long-term maternal outcomes. 
